# Pediatric Challenges With Cowden Syndrome and Graves' Disease: A Case Report

**DOI:** 10.7759/cureus.58090

**Published:** 2024-04-11

**Authors:** Saraladevi Manimaran, Ramya Ramanathan, Sundari Subramanian

**Affiliations:** 1 Department of Pediatrics, Sree Balaji Medical College and Hospital, Chennai, IND

**Keywords:** pediatric patients, pten gene mutations, thyroid disorders, graves' disease, cowden syndrome

## Abstract

Cowden syndrome is a rare genetic anomaly mostly attributed to mutations in the Phosphatase and Tensin (PTEN) Homolog gene. This illness manifests with a diverse array of symptoms that impact several physiological systems and an increased susceptibility to various forms of malignancy. The patient typically exhibits mucocutaneous lesions and a heightened vulnerability to the formation of neoplasms, specifically thyroid carcinomas. The inclusion of thyroid disorders, such as Graves' disease, introduces complications to the management procedure, necessitating a complete approach that includes many healthcare practitioners to guarantee optimal care. Despite some advancements in the field, there remains a dearth of evidence-based recommendations for pediatric patients, encompassing individuals with Cowden syndrome and other thyroid disorders. The current investigation focuses on a 13-year-old female patient who presents with comorbid Cowden syndrome and Graves' disease. We emphasize the challenges associated with the diagnosis and treatment of these illnesses. A collaborative and multidisciplinary team approach was used to administer therapeutic approaches, such as total thyroidectomy, emphasizing the essential requirement for interdisciplinary cooperation among healthcare providers. Continual research endeavors play a pivotal role in elucidating the optimal management protocols and augmenting outcomes for this particular cohort of individuals.

## Introduction

Cowden syndrome is a rare genetic disorder that is inherited dominantly and impacts various bodily systems. The condition is defined by a higher risk of cancer and diverse signs and symptoms, and not all carriers of the genes will manifest the condition. Approximately 85% of cases of this syndrome are primarily due to mutations in the PTEN gene [1]. 15% of mutations are attributed to the following genes: Succinate dehydrogenase (SDH), Phosphatidylinositol-4,5-bisphosphate 3-kinase (PIK3CA), KILLIN (p15INK4b-antisense RNA1, is a non-coding RNA molecule that regulates the expression of the p15 tumor suppressor gene), and AKT1 gene (protein kinase B alpha (PKBα), is a serine/threonine protein kinase involved in multiple cellular processes). The prevalence of Cowden syndrome is approximately one in 200 thousand, with a slight bias towards females, following the elucidation of the genetic pathways implicated [2]. This syndrome tends to appear in individuals aged 20 to 30 and impacts various bodily systems, including the oral mucosa, skin, gastrointestinal tract, thyroid, breast, genital and urinary tract, central nervous system, and bone, with a high tendency to progress into cancer. Initial indications frequently include mucocutaneous manifestations like trichilemmomas, verrucous lesions in the oral cavity, acral keratoses, facial papules, and various benign and malignant tumors, which appear before symptoms affect other areas. Thyroid problems are prevalent in this condition, with some instances presenting adenomatous goiter or thyroid carcinoma.

This emphasizes the critical importance of early detection and treatment. Graves' disease is an autoimmune condition that mainly impacts the thyroid gland, resulting in a swollen thyroid, an overactive thyroid, and eye problems. The precise cause is uncertain, but the formation of GD results from intricate interactions between genetic predisposition and environmental factors. Graves' disease in newborns is rare and is typically treated with antithyroid drugs (ATDs) [3]. Radioiodine ablation or a complete thyroidectomy are considered for cases that do not improve with medication. There is a lack of comprehensive guidelines based on evidence for pediatric patients, particularly those with Cowden syndrome and susceptibility to thyroid disorders, indicating a need for further research. Instances of children having both Graves' disease and Cowden syndrome are extremely uncommon, underscoring the need to enhance comprehension and create tailored treatment approaches for this unique patient population [4]. The optimal treatment strategies for children and adolescents with Cowden syndrome-related thyroid disease remain uncertain despite advancements in medical knowledge. Additional research is required to fill this significant knowledge gap and improve clinical outcomes for affected individuals.

## Case presentation

At the age of 11, a girl who is 13 years old now experienced palpitations, severe anxiety, and intention tremors. She was first given propranolol and hydroxyzine, which led to partial symptom improvement, before being transferred to our hospital for additional assessment. A frameshift mutation in exon 5 of the PTEN gene was identified through genetic testing, confirming the presence of Cowden syndrome. The mutation was also found in her father, who had a total thyroidectomy for a multinodular goiter at the age of 20. Regrettably, he died from a brain tumor at the age of 50. Thyroid cancer also impacted her paternal grandfather, emphasizing the hereditary aspect of the disease. The patient's medical history comprised macrocephaly, a hemangioma in her right lower limb, learning disabilities, and cognitive developmental delay, with an IQ below the reference range. The physical examination showed prominent facial characteristics, a slight protrusion of the jaw, trichilemmomas on the face, and keratoses on the abdomen. An unusual, flexible form of thyroid enlargement was also noted. 

The thyroid function tests revealed notable abnormalities, including thyroid-stimulating hormone (TSH) levels below 0.007 IU/mL, free thyroxine (FT4) levels of 4.94 ng/dL, and free triiodothyronine (FT3) levels of 23.48 pg/mL. Positive anti-thyroid autoantibodies, significantly elevated thyroid peroxidase (TPO) antibodies, and thyroglobulin antibodies suggest autoimmune thyroiditis. An ultrasound of the thyroid showed significant solid nodules with similar echogenicity in both lobes, measuring 12 mm and 100 mm, respectively. The subsequent fine-needle aspiration cytology (FNAC) results were benign and in line with adenomatous/hyperplastic nodules, as shown in Table 1.

**Table 1:** Thyroid Function, Ultrasound, and Surgical Specimen Parameters TSH: Thyroid Stimulating Hormone, FT4: Free Thyroxine, FT3: Free Triiodothyronine, TPO: Thyroid Peroxidase antibodies

Thyroid Function Test Results		
Test	Values	Normal Value
TSH	< 0.007 IU/mL	< 4.0 mIU/L
FT4	4.94 ng/dL	0.7 to 1.8 ng/dL
FT3	23.48 pg/mL	2.3 to 4.2 pg/mL
Anti-thyroid autoantibody	Positiveness	Negative
TPO antibodies	> 3240 IU/mL	< 9 IU/mL
Thyroglobulin antibodies	> 22400 IU/mL	< 4 IU/mL
Thyrotropin receptor antibodies	11.3 IU/mL	Absent or Negative
Thyroid Ultrasound Findings		
	Measurements (mm)	
Right lobe	40 x 84	
Left lobe	49 x 100	
Nodules	12 (right), 100 (left)	
Surgical Specimen		
Surgical Specimen	Weight	
Total Weight	210 g	
Right Lobe	95 mm	
Left Lobe	90 mm	

**Figure 1:**
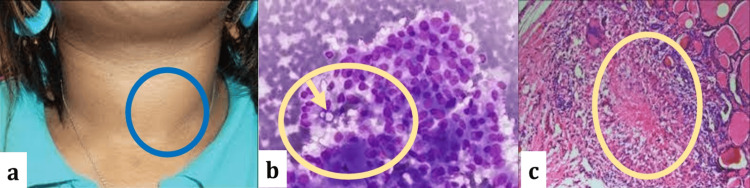
Thyroid Pathological Changes (a) Thyroid Nodule; (b) Violet-Colored Thyroid Cells with Lymphatic Proliferation; (c) Follicular Hyperplasia, Reduction of Colloid, and Patchy Multifocal Lymphatic Proliferations.

A team of specialists, including pediatric and adult endocrinologists, along with a pediatric surgeon, assessed the patient's condition. A complete thyroidectomy was chosen due to the size of the goiter and the risks associated with Cowden syndrome. Before surgery, a potassium iodide-iodine solution (Lugol's) was given to decrease thyroid blood flow and the risk of bleeding during the operation. The surgery was successful, and the specimen weighed 210g, with the right lobe measuring 95 mm and the left lobe measuring 90 mm. Microscopic analysis showed lymphocytic thyroiditis (Figure 1). The patient started taking oral levothyroxine replacement therapy at a dose of 125 µg/day after the surgery to keep their thyroid levels balanced. 

In pediatric patients with Graves' disease, especially those with Cowden syndrome, the initial treatment using propranolol and methimazole was unsuccessful in achieving a normal thyroid function. Follow-up imaging showed significant solid nodules in the thyroid gland, suggesting disease advancement despite medical treatment. A multidisciplinary team suggested a total thyroidectomy because of the large size of the goiter and the risks linked to Cowden syndrome. Surgical intervention was chosen due to the inefficacy of drug treatment, the presence of Cowden syndrome with its cancer risk, and the size of the thyroid nodules. Due to the risks involved with radioiodine ablation, especially in cases of Cowden syndrome, it was decided that surgical removal of the thyroid gland was the best option.

Preoperative preparation involved using Lugol's iodine solution to decrease thyroid vascularity and lower surgical blood loss. The surgery, performed by a pediatric surgeon in conjunction with an adult endocrinologist and a pediatric endocrinologist, went well, leading to the successful removal of the thyroid gland without any issues. Examination of the surgical specimen under a microscope showed lymphocytic thyroiditis, which reinforced the need for surgical treatment. The patient started taking oral levothyroxine replacement therapy at a dose of 125 µg/day after thyroidectomy to keep their thyroid hormone levels balanced. The treatment plan focuses on normalizing thyroid hormone levels and relieving symptoms of hypothyroidism to promote ideal growth and development in adolescence. 

Managing Graves' disease in pediatric patients with Cowden syndrome necessitates a comprehensive strategy that combines medical, surgical, and multidisciplinary interventions. Early identification, consistent observation, and personalized treatment plans are crucial to improving clinical results and reducing the likelihood of complications related to thyroid disease and its treatment in this specific group of patients. Further research and clinical practice will improve treatment guidelines and enhance the quality of care for pediatric patients with Graves' disease and concurrent Cowden syndrome.

## Discussion

The management of Cowden syndrome, especially when Graves' disease is present, emphasizes important factors that need to be considered. Early identification of Cowden syndrome is crucial to starting suitable cancer screening procedures and reducing possible negative consequences linked to the disorder [2]. Due to the common occurrence of thyroid issues in Cowden syndrome, it is advised to undergo regular thyroid evaluations, which should include ultrasound imaging, as part of standard monitoring procedures. 

The NCCN clinical guidelines recommend yearly physical exams, with an emphasis on thyroid assessment, for individuals with Cowden syndrome. The recommendations stress the significance of promptly detecting and monitoring thyroid abnormalities to enable timely intervention and management. Thyroid ultrasound screening is crucial for detecting nodules and structural changes that suggest thyroid pathology [5]. Research indicates that it is recommended to conduct initial thyroid ultrasounds in children diagnosed with Cowden syndrome, followed by annual follow-up examinations. Early thyroid cancer risk in children highlights the need for proactive monitoring to promptly identify and address thyroid issues. Surgical intervention, like a total thyroidectomy, may be necessary to reduce the risk of malignancy and halt disease progression when thyroid nodules are found [6]. 

Graves' disease, an autoimmune thyroid disorder linked to hyperthyroidism, poses distinct challenges for pediatric patients with Cowden syndrome. Radioiodine ablation (RAI) and total thyroidectomy are effective treatments for Graves' disease. However, it is important to consider the potential risks, such as increased cancer susceptibility in individuals with Cowden syndrome. Surgical intervention, specifically total thyroidectomy, may be necessary for certain cases, including individuals with a large goiter, young age, or patient preference [7]. Treating Graves' disease in pediatric patients with Cowden syndrome necessitates a collaborative effort among pediatric endocrinologists, adult endocrinologists, and pediatric surgeons. The size of the goiter, cancer risk related to Cowden syndrome, and patient preferences are crucial factors in deciding the best treatment approach. Before surgical procedures, using Lugol's iodine solution for preoperative preparation can decrease thyroid vascularity and lower the risk of intraoperative complications, such as surgical blood loss [8]. Research on pediatric patients with both Graves' disease and Cowden syndrome is limited. It is crucial to persist in research endeavors to uncover the best management strategies and enhance clinical outcomes for this distinct patient group. Additional research is needed to assess the effectiveness and safety of treatment methods such as surgery and medication in the long term to improve patient care based on solid evidence. Collaboration, continuous monitoring, and patient education are essential in managing the intricate medical requirements of individuals with Cowden syndrome and simultaneous thyroid disorders.

## Conclusions

A multidisciplinary approach is necessary to manage Cowden syndrome and Graves' disease in pediatric patients. Timely identification of Cowden syndrome is essential for starting suitable cancer screening. Regular thyroid evaluations, which include ultrasound imaging, help with prompt intervention. Surgery, like a total thyroidectomy, may be required for cases with large goiters or a high risk of cancer. Effective collaboration among healthcare professionals is crucial for providing the best possible patient care. Additional research is required to enhance treatment protocols and results in this group.
